# Fulvestrant Monotherapy After CDK4/6 Inhibitors in Metastatic Breast Cancer Patients: A Real-Life Experience

**DOI:** 10.3390/cancers16244179

**Published:** 2024-12-15

**Authors:** Margherita Agostini, Anna Mandrioli, Claudio Zamagni

**Affiliations:** 1 Medical Oncology, IRCCS Azienda Ospedaliero-Universitaria di Bologna, 40138 Bologna, Italy; 2 Department of Medical and Surgical Sciences (DIMEC), University of Bologna, 40126 Bologna, Italy; 3 Breast and Gyncological Medical Oncology, IRCCS Azienda Ospedaliero-Universitaria di Bologna, 40138 Bologna, Italy; claudio.zamagni@unibo.it

**Keywords:** fulvestrant monotherapy, CDK4/6 inhibitors, second-line in MBC, progression-free survival

## Abstract

**Background:** The treatment of hormone receptor positive (HR+), HER-2 negative metastatic breast cancer (MBC) has radically changed over the last few years. CDK4/6 inhibitors combined with endocrine therapy have become the standard of care as a front-line therapeutic approach, conferring a significant improvement in progression-free survival and overall survival compared to traditional endocrine therapy (ET) alone. However, the wide administration of these drugs in clinical practice paved the way for the emergence of new intrinsic and acquired mechanisms of resistance that seem to compromise second-line treatment effectiveness. In this context, fulvestrant monotherapy appears disqualified. **Materials and Methods:** we evaluated a population of 30 women currently treated in our oncology unit with HR+/HER2- metastatic breast cancer, receiving fulvestrant 500 mg every 28 days after progression to first-line therapy with CDK 4/6 inhibitors combined with aromatase inhibitors. **Results:** Of 30 patients observed, 23 progressed to fulvestrant with a median PFS of 3.7 months (range 1–9.7 months). **Conclusions:** our real-life experience suggests that second-line fulvestrant monotherapy confers very poor disease control and is quite an inadequate therapeutic option. CDK4/6i administration beyond progression could possibly be considered as more valid option, in the absence of targetable mutations or newer, more effective drugs.

## Simple Summary

The introduction of cyclin-dependent kinases 4 and 6 inhibitors has greatly improved the management of HR+ HER2- metastatic breast cancers, making the first-line approach safe and effective. However, when the disease progresses, the currently available second-line treatments are not as successful. Our study looked at 30 patients who received fulvestrant monotherapy in a second line setting after CDK4/6 inhibitors administration. The results we obtained on survival outcomes were lower than expected.

## 1. Introduction

In recent years, there has been a significant transformation in the treatment approach for managing metastatic breast cancer (MBC), particularly for patients who have hormone receptor-positive (HR+) and human epidermal growth factor receptor 2-negative (HER2-) status. The integration of cyclin-dependent kinases 4 and 6 inhibitors (CDK4/6i), combined with endocrine therapy (ET), has now become the new standard of care (SoC) for first-line treatment in this patient subgroup. This combination therapy rapidly gained prominence as the preferred frontline therapeutic strategy, showing a marked improvement not only in progression-free survival (PFS) but also in overall survival (OS) compared to the conventional approach of endocrine therapy alone [1].

The foundational evidence that led to the approval of CDK4/6 inhibitors initially emerged from the PALOMA-1 trial. This pivotal study compared the combination of palbociclib and letrozole to letrozole alone as a frontline treatment for MBC and reported substantial improvements in progression-free survival (20.2 months vs. 10.2 months) and overall response rate (ORR) (55% vs. 39%) in the palbociclib arm [2]. Additional studies, including MONARCH-2, MONARCH-3, PALOMA-2, PALOMA-3, and MONALEESA-2, 3, and 7, have since validated the survival benefits associated with CDK4/6 inhibitor administration, regardless of whether patients are endocrine-sensitive or resistant [3,4,5,6,7,8]. Concerning overall survival, the first significant data were presented in the MONALEESA-2 trial, which demonstrated that the addition of ribociclib, a CDK4/6 inhibitor, to an aromatase inhibitor (AI) as a frontline therapy substantially increased overall survival (63.9 months vs. 51.4 months, with a hazard ratio (HR) of 0.76; 95% CI, 0.63 to 0.93; *p* = 0.008) [9]. A similar trial, MONARCH-3, evaluated whether adding abemaciclib to AI could also lead to OS improvements. The final results, presented at the American Society of Clinical Oncology (ASCO) 2023, indicated a median OS increase of 13.1 months in the abemaciclib arm; however, despite its clinical significance, this improvement did not reach statistical significance (HR 0.804; 95% CI, 0.6737–1.015; *p* = 0.664) [10].

Beyond survival outcomes, it is important to note that the introduction of CDK4/6 inhibitors into clinical practice has had a transformative impact on the quality of life (QoL) for MBC patients [11].

These agents not only delay the need for chemotherapy, sparing patients from its well-known toxicities and adverse effects, but their oral administration also reduces the need for frequent hospital visits, thereby alleviating patient distress. This dual benefit underscores current guideline recommendations advocating for the early use of CDK4/6 inhibitors in luminal MBC. However, the optimal second-line strategy following CDK4/6 inhibitor failure remains under active discussion, as a universally accepted standard approach has yet to be defined [12].

Since there are no clear guidelines for this setting and the literature lacks sufficiently robust evidence to favor one treatment over another, assessing tumor aggressiveness, sensitivity to hormone therapy, and patient characteristics becomes critically important.

For patients with specific genetic mutations, targeted therapies should be explored, such as alpelisib administration for tumors with PIK3CA mutations and capivasertib for tumors with alterations in the PIK3CA/AKT1/PTEN pathways [13,14]. In cases of highly aggressive tumors or those that progress rapidly after CDK4/6 inhibitors, chemotherapy, typically using anthracycline-based or taxane-based regimens, is often the preferred choice due to its potential for quicker disease control [15]. For tumors classified as endocrine-sensitive, endocrine-based therapies are commonly favored for their efficacy and suitability in less aggressive diseases. Among these, fulvestrant monotherapy is widely regarded as a valid option: it guarantees non-inferior survival outcomes compared to the other strategies, but with a more favorable toxicity profile [15]. If compared with chemotherapies, it substantially avoids bone marrow toxicity, nausea, vomiting, gastrointestinal disturbances, alopecia, and other collateral burdens. At the same time, when compared to other endocrine therapy strategies, such as the combination of mTOR inhibitors like everolimus with endocrine agents like exemestante, fulvestrant monotherapy avoids the metabolic issues, such as hyperglycemia and hyperlipidemia, as well as stomatitis and the fatigue commonly experienced by patients on these regimens. Additionally, fulvestrant monotherapy eliminates the risk of pneumonitis, a less common but potentially severe side effect of everolimus–exemestane treatment [16,17].

**Figure 1:**
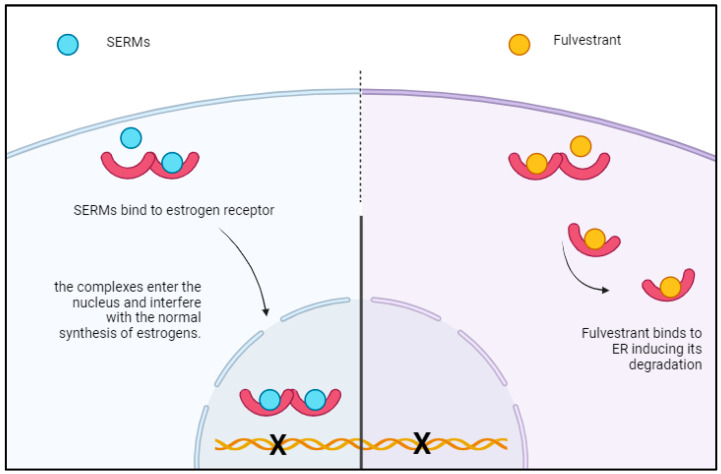
In this figure, a schematic representation of the mechanism of action of fulvestrant compared to traditional SERMs such as tamoxifen.

Fulvestrant functions as a selective estrogen receptor degrader, binding to the estrogen receptor to promote its destabilization, followed by its degradation within the cell [18,19] (Figure 1). It is administered via intramuscular injection once every 28 days, except for an intermediate dose on day 15 during the first month of treatment. Before the advent of CDK4/6 inhibitors, fulvestrant was regarded as one of the most effective endocrine agents for treating luminal MBC, particularly at the higher dosage of 500 mg, owing to its ability to counteract estrogen receptor 1 (ESR1)-mediated hormonal resistance [20,21]. This hormonal resistance remains a critical challenge in the natural history of ER-positive breast cancer.

Since the introduction of CDK4/6 inhibitors in the frontline treatment of luminal MBC, there has been a shift in the treatment paradigm. Although these drugs have been successful, new intrinsic and acquired resistance mechanisms have inevitably surfaced, potentially compromising the effectiveness of traditional second-line treatments [22]. Despite the numerous second-line strategies available for MBC treatment, the results remain unsatisfactory. None of the available options (chemotherapy, hormonal therapy, or targeted therapies) ensure satisfactory disease control in the second-line setting. Literature is increasingly focusing on examining changes in hormonal receptor expression and tumor cell aggressiveness after these treatments.

In this article, we present our real-world experience with fulvestrant administration following disease progression on CDK4/6 inhibitors in patients with ER+ MBC, emphasizing the primary challenges and critical issues that we have encountered.

## 2. Materials and Methods

We conducted an evaluation of a cohort of 30 women who are currently receiving treatment in our oncology unit. Each woman in this group has hormone receptor-positive, HER2-negative metastatic breast cancer (HR+/HER2- MBC) and is undergoing therapy with fulvestrant at a dosage of 500 mg administered through an intramuscular injection every 28 days. This treatment was initiated following disease progression while the patients were receiving CDK4/6 inhibitors combined with a daily aromatase inhibitor (AI) as part of their initial treatment plan. Data collection for this study spanned from 1 January 2020 to 1 April 2023, capturing a comprehensive overview of patient outcomes over this timeframe. The oncological response was assessed approximately every 14 weeks (with a margin of +/− 2 weeks) using various imaging modalities according to routine clinical practice. These included total body CT scans, FDG PET-CT scans, or contrast-enhanced ultrasound (CEUS) of the liver, allowing for a detailed and thorough assessment of treatment efficacy.

## 3. Results

Regarding the cohort’s characteristics, the median age among the participants was 63.8 years, with an age range from 43 to 89 years. Prior to receiving fulvestrant, 16 of these women had been treated with palbociclib, two with abemaciclib, and eleven with ribociclib as part of their frontline CDK4/6 inhibitor therapy. Notably, four women had to switch from one CDK4/6 inhibitor to another during frontline treatment due to issues with drug tolerability, as they experienced toxicity that necessitated a change in medication. Additionally, two patients discontinued their CDK4/6 inhibitor therapy before experiencing disease progression—one due to renal toxicity and the other due to hepatic toxicity.

The median progression-free survival (PFS) duration for patients on CDK4/6 inhibitors was recorded at 22.0 months. Only one patient in the cohort underwent a chemotherapy regimen (specifically, myocet-cyclophosphamide) after progression on CDK4/6 inhibitors but prior to starting fulvestrant, indicating that chemotherapy was generally deferred in favor of continued endocrine therapy in most cases.

In terms of metastatic spread at the time of progression on CDK4/6 inhibitors, twenty women presented with at least one visceral metastasis, four women had only bone metastases, while six others exhibited both lymph node and bone metastases, indicating a mixed metastatic pattern. Detailed cohort characteristics can be found in Table 1, which summarizes the primary clinical and demographic features of the studied population.

**Figure 2:**
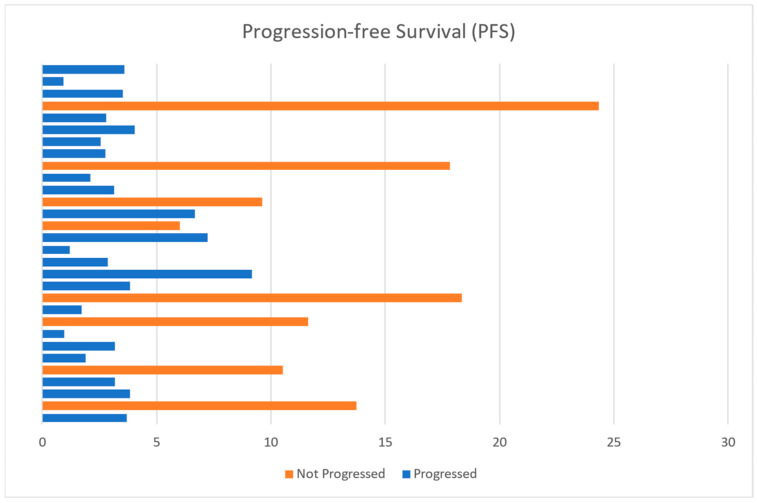
A bar chart demonstrating PFS distribution in the sample. Each horizontal bar represents a patient (*y*-axis), in orange those who did not progress, and in blue those who have progressed. The unit of the *x*-axis is in months.

Among the 30 patients observed in this study, 23 experienced disease progression while on fulvestrant, with a median progression-free survival (PFS) of 3.7 months. The range of PFS in this group varied significantly, spanning from a minimum of 1 month to a maximum of 9.7 months, as illustrated in Figure 2. Notably, the longest PFS (9.7 months) was achieved by an 88-year-old patient, who represents a unique case in our sample. This patient had initially been treated with CDK4/6 inhibitors as part of upfront therapy but had to discontinue the treatment due to renal toxicity. Following this interruption, she continued with letrozole alone until disease progression, at which point she was transitioned to fulvestrant. This sequence of events makes her case an interesting outlier within our population. Conversely, the shortest PFS observed was just 1 month. This rapid progression was detected through an early radiological assessment, which was performed in response to a sudden clinical deterioration in the patient’s condition. The swift onset of symptoms necessitated immediate imaging, which confirmed disease progression after only one month of fulvestrant treatment.

In contrast, six patients in this cohort did not experience disease progression while on fulvestrant therapy at the end of the observation period. Among these, three patients were only recently initiated on fulvestrant monotherapy and thus have not been on the treatment long enough to assess their long-term outcomes. The remaining three patients have shown variable durations of response, with progression-free survival of 5.4 months, 9.5 months, and 16.0 months, respectively. The patient with the longest PFS in this group (16.0 months) was, at the time of the final analysis, our best responder on second-line treatment with fulvestrant. However, it is noteworthy that the latest testing in this patient has revealed a significant increase in carcinoembryonic antigen (CEA) and cancer antigen (CA) 15.3 levels, suggesting a possible approach toward disease progression after a prolonged response to fulvestrant, even though the reliability of these markers in this context remains limited [23].

**Figure 3:**
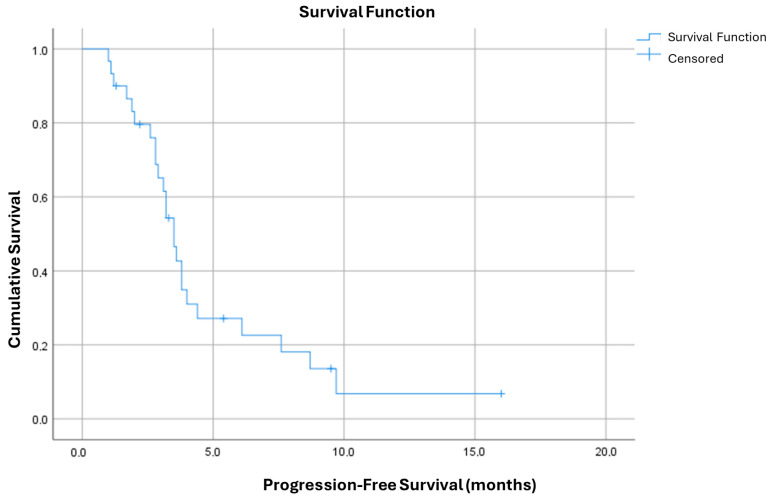
Kaplan Meyer curve for PFS in patients treated with fulvestrant after 1st line therapy with AI + CDK4/6i.

Finally, when examining the cumulative progression-free survival (PFS) for the entire sample of patients, the Kaplan-Meier survival curve reveals a pronounced and rapid decline. Specifically, the curve demonstrates a PFS rate of only 27% at six months, which further drops to a mere 6% at the one-year mark (Figure 3).

## 4. Discussion

Much progress has been made in the last decade concerning the natural history of HR+/HER2- metastatic breast cancer. There is no doubt that CDK4/6i introduction in clinical practice has completely changed patient’s QoL and survival expectations, and this is why current evidence clearly encourages their wide-scale employment; no reserve should be made about risks and benefits balance, which clearly leans towards benefits. The oral administration together with the quite good therapeutic index make these drugs a relatively safe and convenient choice for most of the patients, who manage to live a substantially normal daily routine while affected by advanced disease [24].

On the contrary, in our second-line real life experience, fulvestrant monotherapy clearly confers very poor survival outcomes and based on these results can be considered an unsatisfying therapeutic option. Among our cohort of 30 patients, we observed a median PFS of only 3.7 months on fulvestrant, with a steep decline in survival benefits as indicated by the Kaplan-Meier curve, which revealed a mere 27% PFS rate at six months and only 6% at one year. Our data also show considerable variability in patient responses, from cases of rapid progression within the first month to outliers who achieved up to 9.7 months of PFS. Despite this variability, the overall survival outcomes remained poor, underscoring that fulvestrant alone is insufficient as a second-line strategy following CDK4/6i progression.

A wide range of responses was noted, with PFS ranging from as short as 1 month to a maximum of 9.7 months. This variability highlights the heterogeneous nature of the patient population and the impact of individual factors, such as prior CDK4/6i experience, patient age, and specific comorbidities. Notably, the longest PFS was achieved by a patient who had an unusual treatment trajectory—she discontinued CDK4/6i therapy due to renal toxicity and continued with letrozole alone until progression. Her longer response suggests that disease- and patient-specific factors can significantly influence outcomes, warranting further investigation into personalized treatment approaches post-CDK4/6i progression.

Several new ET are currently under clinical evaluation, and among those, new oral selective estrogen receptor degraders (SERD) seem to be a promising alternative to fulvestrant, representing its natural upgrading. On this purpose, elacestrant is the first oral SERD tested in a phase III clinical study (EMERALD) as second-line option beyond CDK4/6i progression compared to standard of care endocrine monotherapy (including fulvestrant monotherapy). Results show that elacestrant significantly prolonged progression-free survival (PFS), particularly in MBC patients with ESR1 mutations. The hazard ratios (HR) for PFS in the overall population and in patients with ESR1 mutations were 0.70 and 0.55, respectively, demonstrating a reduction in the risk of progression or death by 30% and 45% for elacestrant subgroup. Additionally, treatment-related adverse events, such as nausea, were more frequent with elacestrant but generally manageable, with grade 3/4 adverse events reported in only 7.2% of patients. This study establishes elacestrant as a promising oral alternative to intramuscular fulvestrant, offering a new, effective treatment option for this patient population with an acceptable safety profile [25]. With interesting subgroup analysis and a real-world experience which were presented at ASCO 2023 and ASCO 2024, respectively, elacestrant received European Medical Agency (EMA) approval in a second-line setting for ESR1 mutated, hormone positive, HER2 negative MBC. Kaklamani particularly focused on patients who had rapidly progressed within six months of prior CDK4/6i therapy, revealing that elacestrant significantly prolonged progression-free survival (PFS) in this subgroup, with a median PFS of 5.32 months versus 1.87 months for SoC. This benefit was less pronounced in patients with longer durations of prior CDK4/6i therapy. Additionally, elacestrant was found to extend the time to chemotherapy compared to SoC, consistent with preclinical evidence of its activity in ESR1 wild-type tumors. Lloyd’s data provide real-world insights into the use patterns and therapeutic outcomes of elacestrant. Utilizing the GuardantINFORM database, it examined 418 patients treated after FDA approval, assessing metrics such as time to treatment discontinuation (TTD) and time to next treatment (TTNT) as proxies for progression-free survival (PFS). The findings reveal median TTD and TTNT values of 5.4 and 6.2 months, respectively, exceeding the median PFS of 3.8 months reported in the EMERALD trial. Interestingly, the number of prior lines of therapy or ESR1 mutations did not significantly influence these outcomes. The discrepancies between real-world and clinical trial results may reflect differences in patient selection and care protocols [26,27,28].

An ongoing phase III trial, VERITAC 2 (NCT05654623), is currently evaluating the efficacy of ARV-471 (vepdegestrant), a proteolysis targeting chimera estrogen receptor degrader (PROTAC), compared to fulvestrant in patients who progressed after first-line CDK4/6 inhibitor therapy [29]. Vepdegestrant is a potent, selective, and orally bioavailable small molecule that binds the intracellular E3 ligase cereblon (CRBN) and the ligand-binding domain (LBD) of the estrogen receptor (ER), facilitating the rapid degradation of ER via the cell’s ubiquitin-proteasome system (UPS). Preclinical studies demonstrated that vepdegestrant selectively degraded more than 80% of ER within 4 h across multiple ER+ cell lines, including ligand-independent ER mutants, and achieved over 90% degradation in vivo models. This is significantly superior to fulvestrant, which degraded only 40%–60% of ER, aligning with observations in patients. These differences also translated into considerably improved tumor growth inhibition with vepdegestrant compared to fulvestrant, supporting its progression to clinical trials for ER+/HER2- advanced breast cancer [30].

In the context of managing hormone receptor-positive (HR+), HER2-low, or HER2-ultralow metastatic breast cancer (mBC) after rapid progression on CDK4/6 inhibitors combined with endocrine therapy (ET), one such option is trastuzumab deruxtecan (T-DXd), an antibody-drug conjugate shown to provide significant clinical benefits in this setting. Data from the DESTINY-Breast06 trial demonstrate that T-DXd offers a marked improvement in progression-free survival (PFS), with a median of 13.2 months compared to 8.1 months with chemotherapy in the HER2-low subgroup (HR 0.62; 95% CI: 0.51–0.74; *p* < 0.0001). Notably, this subgroup included patients who experienced disease progression within six months of CDK4/6 inhibitors plus ET, underscoring the efficacy of T-DXd in overcoming resistance to prior therapies. Furthermore, the trial reported an overall response rate (ORR) of 56.5% for T-DXd versus 32.2% for chemotherapy, highlighting its potential to achieve durable responses. While safety considerations such as interstitial lung disease must be weighed, the compelling efficacy data support the integration of T-DXd as a valuable option in this challenging treatment scenario [31].

Another debated option for the second-line setting is the continuation of CDK4/6i therapy beyond progression: in this regard, the ongoing phase-II MAINTAIN trial has demonstrated that the combination of ribociclib and traditional endocrine second-line therapy (fulvestrant or exemestane) after previous CDK4/6i progression (85% Palbociclib) significantly improves mPFS if compared to endocrine monotherapy alone (5.33 vs. 2.76 months, HR 0.56; 95% CI 0.37–0.8 *p* = 0.004) [32]. On the contrary, contrasting evidence come from the PACE trial in which Palbociclib administration after previous CDK4/6 progression (mainly palbociclib) seems not to improve PFS [33].

Anyhow, real-life experience suggests survival benefits for CDK4/6i continuation: J. Martin et al. analysed a population of 839 patients who received a second-line systemic therapy which was chosen case-by-case among chemotherapy, endocrine monotherapy, targeted therapies when possible (such as everolimus, alpesilib or Poly ADP-ribose polymerase (PARP) inhibitors), and second-line setting CDK4/6i; it was demonstrated that patients continuing CDK4/6i had significantly better outcomes than those who received chemotherapy both in terms of PFS (8.25 vs. 3.71 months HR 0.48, 95% CI 0.43–0.53, *p* < 0.0001) and of OS (HR 0.30, 95% CI 0.26–0.35, *p* < 0.0001), regardless whether the second-line CDK4/6 inhibitor was the same of the up-front treatment or not [34]. The authors demonstrated that fulvestrant monotherapy was not superior to chemotherapy-based regimen neither in terms of PFS (3.25 vs. 3.71 months) nor of OS, while everolimus overcame chemotherapy only for OS. These data suggest that the continuation of the CDK4/6i after first-line progression should be considered as a valid therapeutic option, better if combined to ET-partner switching, in selected patients. While some positive considerations might be made even for everolimus second-line administration, fulvestrant monotherapy appears disqualified.

## 5. Conclusions

The molecular mechanisms’ underlying acquired resistance to CDK4/6 inhibitor-based treatment in MBC remain poorly understood, and real-world data clearly indicate that second-line fulvestrant monotherapy achieves suboptimal disease control. This underscores the urgent need for novel therapeutic strategies, several of which are currently being evaluated in clinical trials. Among these, elacestrant has shown modest benefits in managing progressed HR+/HER2- MBC; however, its use is presently limited to patients with ESR1 mutations as per EMA approval. Other promising therapeutic options are emerging, but they are not yet globally available for clinical application. In the absence of targetable mutations and in selected ESR1 wild-type patients, particularly those with asymptomatic disease progression and low tumor burden, continuing CDK4/6 inhibitors beyond progression may represent a more viable approach compared to fulvestrant monotherapy in the second-line setting. Until more robust evidence becomes available, close clinical and radiological monitoring of cancer response during fulvestrant therapy as part of the standard of care remains a prudent recommendation.

## Figures and Tables

**Table 1:** Patients Characteristics.

Variable	Sample Assessment		Average Values
Age	<60	15	63
	>60	18	
Front-line CDK4/6 inhibitor	Palbociclib	16	
	Abemaciclib	2	
	Ribociclib	11	
	Unknown	1	
Median PFS to CDK4/6 inhibitor	Palbociclib	25.4	22.0
	Abemaciclib	-	
	Ribociclib	19.8	
	Unknown	-	
Dose reduction need during CDK4/6i	None	4	
	At least one	26	
	Personalised schedule	3	
CDK4/6i interruption for toxicity	Palbociclib	0	
	Abemaciclib	1 (renal toxicity)	
	Ribociclib	1 (hepatic toxicity)	
CDK4/6i switch before progression	Palbociclib	2 (switch to Abemaciclib)	
	Abemaciclib	0	
	Ribociclib	3 (switch to Palbociclib)	
Sites of metastasis at CDK4/6i progression (before fulvestrant administration)	Bones only	4	
	Lymph nodes or subcutaneous tissue (in addition to bones or not)	6	
	At least one visceral metastasis	20	

## Data Availability

The datasets generated during and/or analyzed during the current study are available from the corresponding author on reasonable request.
